# Amplification-Free Detection of Highly Structured RNA Molecules Using SCas12aV2

**DOI:** 10.21769/BioProtoc.5462

**Published:** 2026-06-20

**Authors:** Teng Hu, Youyang Pei, Zhaoyi Hu, Jing Feng, Qiangyuan Jiang, Li Hu, Yi Liu

**Affiliations:** 1 Hubei Light Industry Technology Institute, Hubei, China; 2 State Key Laboratory of Biocatalysis and Enzyme Engineering, School of Life Sciences, Hubei University, Hubei, China

**Keywords:** CRISPR/Cas12a, crRNA, Spacer RNA, Scaffold RNA, SCas12a system

## Abstract

The CRISPR/Cas12a system has revolutionized molecular diagnostics; however, conventional Cas12a-based methods for RNA detection typically require transcription and pre-amplification steps. Our group has recently developed a diagnostic technique known as the SCas12a assay, which combines Cas12a with a split crRNA, achieving ampliﬁcation-free detection of miRNA. However, this method still encounters challenges in accurately quantifying long RNA molecules with complex secondary structures. Here, we report an enhanced version termed SCas12aV2 (split-crRNA Cas12a version 2 system), which enables direct detection of RNA molecules without sequence limitation while demonstrating high specificity in single-nucleotide polymorphism (SNP) applications. We describe the general procedure for preparing the SCas12a system and its application in detecting RNA targets from clinical samples.

Key features

• The SCas12aV2 assay enables efficient detection of long-chain RNA molecules with complex secondary structures.

• PAM-distal sites can be effectively distinguished at the SNP detection level.

• The entire experimental procedure can be completed in less than one hour.

## Background

In recent years, novel nucleic acid detection technologies, such as CRISPR/Cas12a, have been successfully applied in various fields, including viral identification, disease diagnosis, and prognostic assessment. Cas12a has two cleavage activities upon target recognition: (i) *cis*-cleavage, which cuts target double-stranded DNA (dsDNA) when a matching sequence is present in the crRNA spacer, and (ii) *trans*-cleavage, which degrades nearby single-stranded DNA (ssDNA) nonspecifically after activation. This trans-cleavage activity enables highly sensitive nucleic acid detection methods like DETECTR [1] and HOLMES [2], demonstrating their value in point-of-care diagnostics.

**Figure 1.**
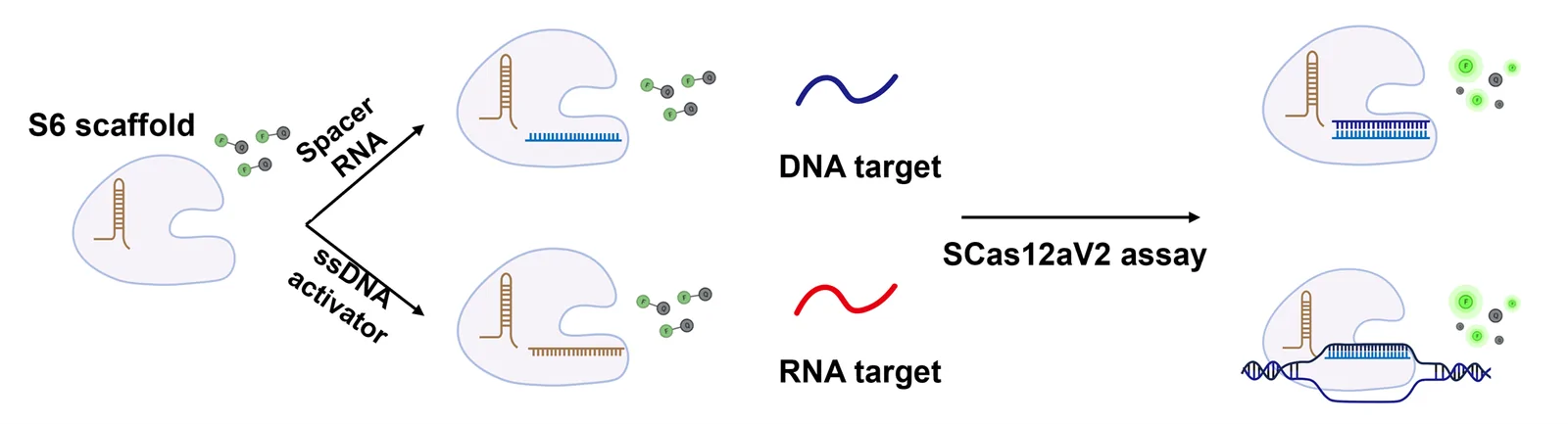
Schematic representation of the SCas12a assay for nucleic acid detection

To date, most Cas12a-based methods [1,2] still require integration with nucleic acid amplification techniques to achieve detection efficiencies comparable to those of qPCR. Moreover, these methods are incapable of directly quantifying RNA targets. Recently, we discovered that crRNA can be divided into two fragments: scaffold RNA and spacer RNA. These fragments subsequently reassemble with the Cas12a protein to form a ribonucleoprotein (RNP) with *trans*-cleavage activity. Based on this discovery, we developed a split-crRNA Cas12a (SCas12a) system [3] for point-of-care testing (POCT) RNA detection technologies (Figure 1), thereby eliminating the need for reverse transcription and nucleic acid amplification. This approach achieves an average limit of detection (LoD) value of 100 fM for miRNA by fluorescence assay and of 10 fM for miRNA by lateral flow assay (LFA). Furthermore, we developed an enhanced version of the method, termed SCas12aV2 [4], which enables efficient detection of RNA substrates with long sequences and complex structures. The assay is highly versatile and can be readily adapted to various Cas12a orthologs, thus advancing molecular diagnostics by improving the accuracy and efficiency of RNA detection. Collectively, this method can be applied in various settings, such as the detection of RNA from epidemic pathogens and the identification of RNA-based cancer markers from clinical samples, facilitating rapid mutant screening for breeding purposes.

## Materials and reagents

**Reagents**

1. 10× PCR buffer (with Mg^2+^) (Beyotime, catalog number: D7223-1)

2. 10× NEBuffer r2.1 (NEB, catalog number: B6002S)

3. 1 M Tris pH 8.0, sterile (Sangon Biotech, catalog number: B548127-0500)

4. Sodium chloride (NaCl) (Sangon Biotech, catalog number: A610476-0001)

5. DTT (Sangon Biotech, catalog number: A620058-0100)

6. HOLMES-Fluo ssDNA reporter 1 (FAM) (TOLOBIO, catalog number: 31101), 5’FAM-TTATTATT-3’BHQ1

7. AsCas12a protein: Synthetic genes encoding the CRISPR-associated protein AsCas12a were cloned into the pET-28a(+) expression vector to construct plasmids for recombinant protein production. The sequence accuracy of these plasmids was verified prior to transformation into *Escherichia coli* BL21 (DE3) competent cells. For protein expression, a single colony was inoculated into LB broth supplemented with ampicillin (100 μg/mL) and incubated overnight. The following day, these cultures were diluted into 1 L of Terrific Broth (TB) medium to an optical density at 600 nm (OD_600_) of 0.8, cooled on ice for 10 min, induced with 0.5 mM isopropyl β-D-1-thiogalactopyranoside (IPTG), and further incubated at 18 °C for 16 h. The bacterial cells were then pelleted by centrifugation and lysed in buffer A [20 mM Tris-HCl, pH 7.5, 300 mM NaCl, 1 mM phenylmethanesulfonyl fluoride (PMSF), 5 mM β-mercaptoethanol] containing a protease inhibitor cocktail. The recombinant Cas proteins were purified using immobilized metal affinity chromatography (IMAC) with Ni-NTA resin, followed by size-exclusion chromatography on a HiLoad^®^ 16/600 Superdex^®^ column according to a previously published protocol [5]. The purified proteins were concentrated using a 100 kDa molecular weight cutoff (MWCO) centrifugal filter, and their concentrations were determined by the Bradford assay. Subsequently, the concentrated proteins were flash-frozen in liquid nitrogen and stored at -80 °C for future use.

**Solutions**

1. 10× PCR buffer (with Mg^2+^) (see Recipes)

2. 10× NEBuffer r2.1 (see Recipes)

3. Storage buffer (see Recipes)

**Recipes**

**1. 10× PCR buffer (with Mg^2+^)**

100 mM Tris-HCl (pH 8.8 at 25 °C)

500 mM KCl

15 mM MgCl_2_

0.8% (v/v) Nonidet P40

**2. 10× NEBuffer r2.1**

500 mM NaCl100 mM Tris-HCl100 mM MgCl_2_100 μg/mL recombinant albuminpH 7.9 at 25 °C

**3. Storage buffer**

20 mM Tris-HCl

250 mM NaCl

1 mM DTT

pH 7.9 at 25 °C

**Laboratory supplies**

1. Pipette tips (filtered) (Thermo, catalog numbers: TF112-1000-Q, T104RS-Q, TF140-200-Q)

2. 0.2 mL PCR tubes (Thermo, catalog number: 431-MIXED-Q)

3. 1.5 mL microtubes (AXYGEN, catalog number: MCT-150-L-C)

## Equipment

1. Real-time PCR system (Bio-Rad, model: CFX96 touch, or Stratagene^TM^ Mx3005P, Thermo, model: PF1457N)

2. Microcentrifuge (mySPIN^TM^ 6 Mini, Thermo, model: 75004061 or equivalent)

3. Thermal cycler (VeritiPro PCR, Thermo, model: A48141)

4. Vortex mixer (Eppendorf mixer, Eppendorf, model: 5382000074)

5. Variable volume pipettes (P10, P200, P1000)

6. Freezer (-80 °C) (Thermo, model: 905)

7. Standard laboratory equipment (analytical balance, pH meter, etc.)

## Software and datasets

1. RNA secondary structure prediction software is listed as follows:

NUPACK: http://www.nupack.org/

RNAfold: http://rna.tbi.univie.ac.at/cgi-bin/RNAWebSuite/RNAfold.cgi

Mfold: http://unafold.rna.albany.edu/?q=mfold

**Figure 2.**
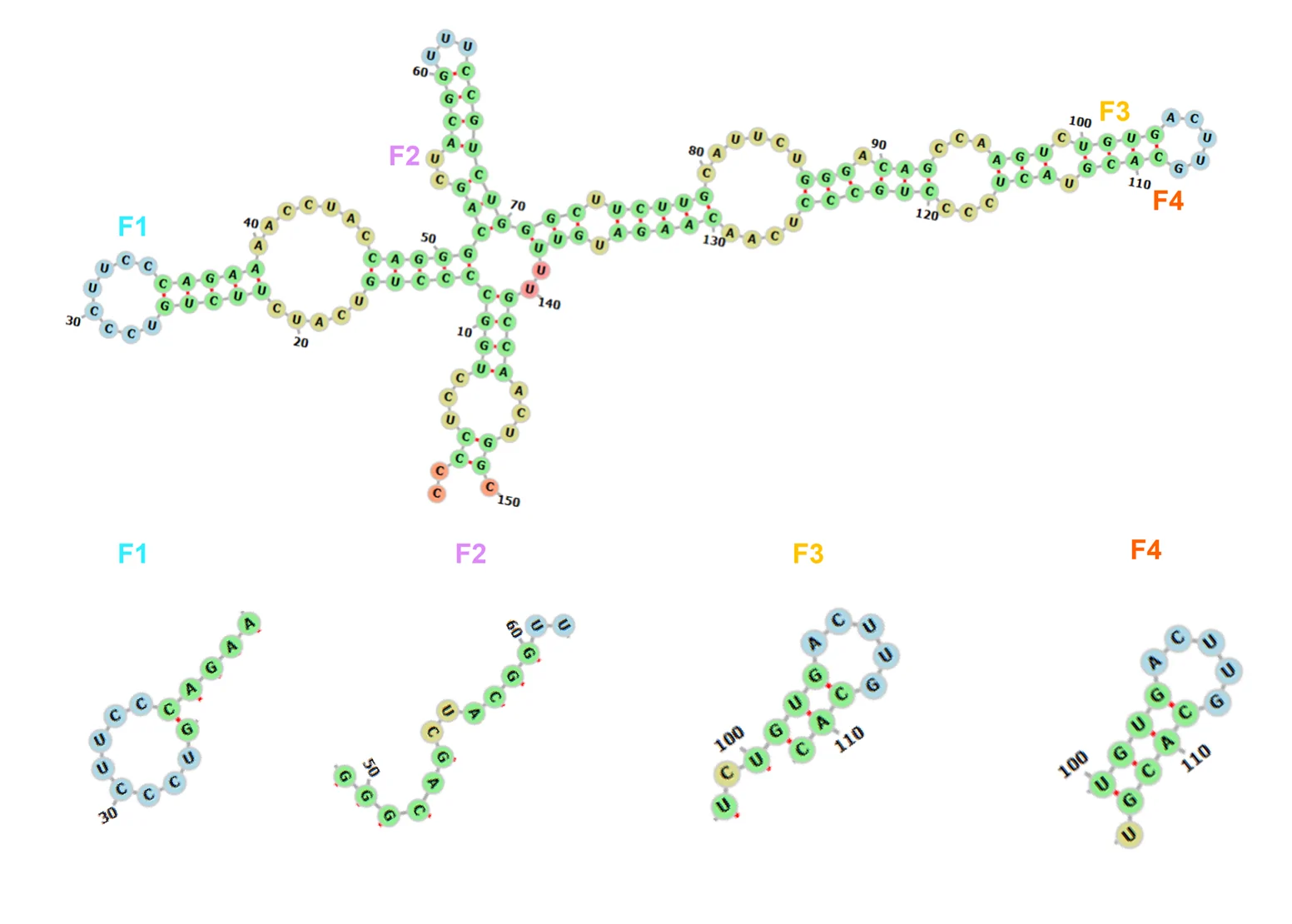
Schematic diagram of the structure predicted by NUPACK for the P53 RNA

For the prediction of highly structured RNA regions:

1. Secondary structure prediction: Utilize NUPACK or RNAfold to identify regions with low free energy (Figure 2).

2. Target site selection: We used NUPACK to predict the secondary structures and free energy distribution of the target RNA. The results demonstrated that regions with higher free energy exhibit lower thermodynamic stability, rendering them more susceptible to disruption by activators and thus more suitable as target sites.

**Figure 3.**
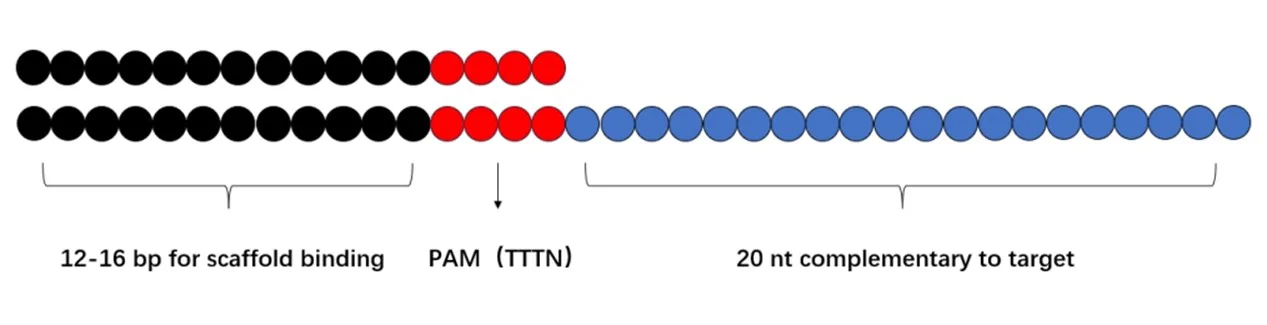
Schematic diagram of hybrid activator design

For the design of the dsDNA-ssDNA hybrid activators: The hybrid activator improves detection efficiency and sensitivity compared to traditional ssDNA or dsDNA activators by enabling stronger and more stable interactions between the RNA substrate and the Cas12a active site. The design principle is outlined in the following:

1. A double-stranded DNA region: 12–16 bp for scaffold binding (Figure 3).

2. PAM sequence: TTTN (N = A, C, or G).

3. A single-stranded DNA region: 20 nt complementary to the target.

## Procedure

**A. Prepare the SCas12aV2 system for RNA detection**

1. Prepare dsDNA-ssDNA hybrid activators in a 0.2 mL PCR tube.

a. Synthesize the primers for constructing the dsDNA-ssDNA hybrid activators using the services of Sangong Biotech Co. b. Add 1 μL each of forward and reverse primers from a 10 μM stock solution. c. Add 1 μL of 10× PCR buffer. d. Add 7 μL of ddH2O (the total reaction volume is 10 μL, resulting in a final primer concentration of 1 μM). e. Place the tube in the PCR machine and set the annealing protocol as follows: gradually decrease the temperature from 95 °C to 25 °C at a cooling rate of 0.1 °C/s. f. Dilute the dsDNA-ssDNA hybrid activators to 500 nM and store them for subsequent use.

**B. Prepare the SCas12a fluorescence assay in a 0.2 mL PCR tube**

1. Dilute AsCas12a to 2.5 μM using storage buffer and scaffold RNA to 5 μM using ddH_2_O and store them for subsequent use. (The protein concentration can be adjusted to 5 μM and the scaffold RNA concentration to 10 μM, depending on the protein activity.)

2. Add 1 μL of 10× NEB buffer r2.1.

3. Add 1 μL of AsCas12a protein (2.5 μM).

4. Add 1 μL of scaffold RNA (5 μM).

5. Incubate the mixture at 37 °C for 10–15 min to allow RNP complex formation.

6. Add 4 μL of ddH_2_O.

7. Add 1 μL of dsDNA-ssDNA hybrid activators from the previously prepared 500 nM stock solution.

8. Add 1 μL of FQ reporter from a 10 μM stock solution.

9. Add 1 μL of RNA target from a 100 nM stock solution.

10. Quickly seal the PCR tube lid and mix the contents by brief centrifugation.

11. Detect the target on the CFX96 Touch Real-Time PCR System, incubating the reaction at 37 °C for 60 min while monitoring fluorescence emission at 520 nm.

## Data analysis

To determine the exact concentration of an RNA, it is essential to first establish a linear correlation between fluorescence intensity and the concentration of RNA standards. Detailed information can be found in our previous study [4]. In that work, we have successfully established a linear correlation between the ﬂuorescence intensity and the concentration of TP53 RNA standards (Supplementary Figure S5 in [4]).

## Validation of protocol

This protocol (or parts of it) has been used and validated in the following research articles:

- Chen et al. [3]. Split crRNA with CRISPR-Cas12a enabling highly sensitive and multiplexed detection of RNA and DNA. *Nat Commun.*
- Zhang et al. [4]. Precise ampliﬁcation-free detection of highly structured RNA with an enhanced SCas12a assay. *Commun Biol.*
  Specifically, the quantification of an RNA target using a standard curve can also be found in these manuscripts.

## General notes and troubleshooting

**General notes**

1. The secondary structures and local free energy distribution of the long-chain RNA should be predicted first using appropriate software, such as NUPACK and RNAfold, followed by selecting regions with higher free energy as target sites.

2. Pooled DNA activators should be utilized to achieve high sensitivity at the attomolar level.

**Troubleshooting**

Problem 1: The fluorescence intensity is too low.

Possible causes: The concentration of the target RNA is insufficient, or the RNA has undergone degradation.

Solutions: Increase the amount of high-quality RNA by collecting additional clinical samples or optimizing RNA extraction protocols.

Problem 2: The background fluorescence intensity is too high.

Possible causes: Reduced activity of the Cas12a protein due to protein storage, contamination, and RNA secondary structure effects that may interfere with assay performance.

Solutions: Use freshly prepared Cas12a protein for experiments and modify the target RNA sequences to minimize interference.
